# In vitro cholesterol lowering activity of *Ganoderma australe* mycelia based on mass spectrometry, synchrotron Fourier-transform infrared analysis and liver-spheroid bioactivity

**DOI:** 10.1038/s41598-023-40861-8

**Published:** 2023-08-21

**Authors:** Sudthirak Wongkhieo, Wanthongchai Tangmesupphaisan, Jeeraprapa Siriwaseree, Yaovapa Aramsirirujiwet, Prissana Wiriyajitsomboon, Tharnrat Kaewgrajang, Saifa Pumloifa, Atchara Paemanee, Buabarn Kuaprasert, Kiattawee Choowongkomon, Adrian H. Chester, Napachanok M. Swainson

**Affiliations:** 1 Department of Biochemistry, Faculty of Science, Kasetsart University, Bangkok, 10900 Thailand; 2 Department of Microbiology, Faculty of Science, Kasetsart University, Bangkok, 10900 Thailand; 3 Department of Forest Biology, Faculty of Forestry, Kasetsart University, 50 Ngamwongwan Rd, Lat Yao, Chatuchak, Bangkok, 10900 Thailand; 4 National Center for Genetic Engineering and Biotechnology (BIOTEC), National Science and Technology Development Agency (NSTDA), Pathum Thani, 12120 Thailand; 5 Research Facility Department, Synchrotron Light Research Institute (Public Organization), 111 University Avenue, Muang District, Nakhon Ratchasima, 30000 Thailand; 6 Heart Science Centre, Magdi Yacoub Institute, Harefield, UK; 7 National Heart and Lung Institute (NHLI), Imperial College London, London, UK

**Keywords:** Biochemistry, Microbiology

## Abstract

Mycelia were cultivated from a Thai wild mushroom identified as *Ganoderma australe* based on polymerase chain reaction (PCR) and morphological analyses*.* The mycelial extracts were examined for their active ingredients using a liquid chromatography-tandem mass spectrometry (LC‒MS/MS) method. This revealed the presence of lovastatin and tentative compounds including *p*-coumaric, nicotinamide, gamma-aminobutyric acid, choline, nucleosides, amino acids, and saccharides. The extracts had an inhibitory effect on the activity of HMG-CoA reductase in a concentration-dependent manner. At 2.5 mg/mL, the *G. australe* extracts did not interfere with the viability of HepG2 spheroids, but their biochemical composition was altered as determined by Fourier-transform infrared (FTIR) spectroscopy. The lipid profile of the spheroids treated with the mycelial extract was distinct from that of the control and the 5 µM lovastatin treatment, corresponding with the production of cholesterol by the spheroids. The mycelia of *G. australe* increased the percentage of high-density lipoprotein (HDL) production to 71.35 ± 2.74%, compared to the control and lovastatin-treated spheroids (33.26 ± 3.15% and 32.13 ± 3.24%, respectively). This study revealed the superior effect of natural compound mixtures to pure lovastatin, and the potential use of Thailand’s wild *G. australe* as a functional food to prevent or alleviate hypercholesterolemia.

## Introduction

Hypercholesterolemia is one of the major risk factors for cardiovascular disease^1^ which was the 8th most important risk factor for global mortality in 2019^2^. Statins are a group of drugs prescribed for the treatment of high plasma cholesterol and cardiovascular disease^3^; however, their long-term consumption is not recommended because of their side effects on muscles^4^. This makes alternative treatment using nutraceuticals a promising option^5^, including using mushrooms as functional foods^6^, due to the presence of bioactive compounds. Furthermore, there is an increasing amount of evidence showing that edible mushrooms improve lipid profiles and reduce the risk of cardiovascular disease^7^.

*Ganoderma* is a genus of fungi in the Ganodermataceae family and one of the most important medicinal fungi for humans worldwide^8^. The *Garnoderma* genus contains various species that have been used for medical treatments, including anticancer^9^ diabetes^10^ immunomodulation^11^, and cardiovascular and metabolic disease^12^ treatment properties. The bioactive compounds associated with such medical functions include triterpenoids, polysaccharides, sterol and alkaloids^13^. While the biodiversity of fungi in Thailand has been studied^14^, the identification of a mushroom naturally grown in Thailand with the potential function of hypercholesterolemia treatment has not yet been reported.

In this regard, a wild mushroom with the morphology of *Ganoderma* spp. was collected, cultured for its mycelia and identified at the species level. Lovastatin and biological components of the ethanol-extracted mycelia were identified using high-resolution mass spectrometry (HRMS). Its potential use for the treatment of hypercholesterolemia was investigated based on the inhibitory activity of HMG-CoA reductase. In addition, the cytotoxicity, characteristic functional groups (using FTIR) and cholesterol production (based on 3-D liver cells) in response to the *Ganoderma australe* mycelial extract were elucidated.

## Methods

### Mycelial cultures and molecular identification

Wild mushrooms were collected from the Trat Agroforestry Research and Training Station, Trat Province, Thailand (12° 23′ 34″ N, 102° 40′ 32″ E). The collection of the mushroom and the performance of related experimental research complied with the national guidelines of Thailand. Fruiting bodies were carefully taken from the substrate. Their morphological characteristics were identified and described following Luangharn et al.^15^. A voucher specimen of this material was deposited for public use at Bangkok Forest Herbarium (BKF), Thailand, as T. Kaewgrajang & S. Mangkalad G1-09102018 (BKF, dry and spirit collections).

To obtain culture, the inside uncontaminated tissue of the mushroom specimen was isolated on potato dextrose agar (PDA). Then, mycelia growing out of the transferred mushroom pieces without contamination were subcultured onto new agar media. Pure cultured mycelia were cultivated on PDA covered with cellophane for 7–10 days at room temperature (25–33 °C) before the fungal species was investigated and bioactive component extraction was performed using approximately 200 plates per batch.

The genetic identity of *Ganoderma australe* was confirmed using the internal transcribed spacer (ITS) region covering 18S, ITS1, 5.8S, ITS2 and part of the 28S rDNA. The ITS region was amplified using the primers ITS1 (5′-TCCGTAGGTGAACCTGCGG-3′) and ITS4 (5′-TCCTCCGCTTATTGATATGC-3′) and previously described PCR conditions^16^. Then, nucleotide sequences were analyzed using the MiSeq System (Illumina) with NGS-based barcode taq sequencing (BTSeq™). Species identity was performed based on a database search using the BLASTN program^17^ in the NCBI database, and the best match sequences were retrieved from the database for phylogenetic analysis together with sequences from the current study. The sequence determined in the current study was deposited in the NCBI database under accession number OP727592.

### Ethanol extraction

The mycelia were removed from the culture plates, dried at 50 °C for 5 h and ground using a mortar and pestle. One hundred milligrams of dried mycelia were mixed with 1 mL of 95% ethanol and shaken at 180 rounds per minute at 37 °C for 24 h. The extracted compound was collected after cold centrifugation at 8000 RPM for 15 min and filtered through Whatman No. 1 paper. The supernatant was placed in a hot-air oven at 60 °C to remove any ethanol. The dried extracts were weighed and then dissolved in dimethyl sulfoxide (DMSO) for further analysis.

### High performance liquid chromatography analysis of mycelial extracts and lovastatin

The dried extract was reconstituted in DMSO, diluted with ethanol and filtered before analysis using a high-performance liquid chromatography (HPLC) analyzer (Shimadzu and Hitachi) in the reverse-phase with a C18 column (TOSOH). The separation process was carried out using buffer A (0.1% trifluoroacetic acid (TFA) and buffer B (100% acetonitrile and 0.1% TFA) as the mobile phase. At a constant flow rate of 1 mL/min, buffer B was increased to 40% within 4 min and then slowly increased to 44% during the next 8 min before rapidly increasing to 64% for 2 min. It slowly increased to 68% for 8 min during the expected retention time of lovastatin. The process was finished by increasing buffer B to 100% for 2 min and maintaining for 4 min before reconditioning the column with 100% buffer A for 5 min. The profile was determined using a UV analyzer at *λ* = 240 nm. Data were acquired using the Primaide System Manager software. The chromatographic analysis was carried out for the lovastatin standard at various concentrations (range 0.625–20 µM), the mycelial extracts at 10 µg/mL from 3 different cultured and lovastatin-spiked extracts containing final concentrations of 20 µM lovastatin and 8 µg/mL extract. The amount of lovastatin standard was calculated from the calibration curve of the area of the peak at a retention time of 19.0 min. The limit of detection (LOD) was calculated from the standard curve using the formula LOD = 3.3 × (σ/s) where σ is the SD of the intercept and s is the slope of the curve.

### Liquid chromatography‒mass spectrometry analysis

HPLC fractions with the same retention time as lovastatin were collected from 3.2 mg of crude extract, subjected to mass spectrometry analysis (micrOTOF-Q III) with electrospray ionization and operated in positive polarity mode under the following conditions: m/z range 50–600; capillary voltage, 4500 V; end plate offset voltage, − 500 V; collision cell RF, 100.0 Vpp; nebulizer, 0.3 bar; heater temperature, 180 °C; and dry gas flow rate, 4.0 L/min. The presence of lovastatin in the fraction was further confirmed using MS/MS (SCIEX, X500R QTOF) in multiple reaction monitoring (MRM) mode to acquire mass spectra. The target precursor ion value of lovastatin was 405.26 Da and was ionized using an ion source gas (20 psi), collision gas (7 psi) and curtain gas (25 psi). The time of flight of the ions was operated in positive polarity mode under the following conditions: *m/z* scan range, 100–450; spray voltage, 5500 V; declustering potential, 80 V; and accumulation time, 0.1 s. The isotopic and mass spectral patterns were compared to blank solvent and the lovastatin standard.

The biocomponents in the ethanol extract of mycelia were analyzed using LC‒MS/MS at NSTDA, Thailand, according to the reported protocol^18^. Dried extracts were reconstituted in methanol and filtered through a 0.22 µm PTFE membrane before chromatographic separation was carried out. Each sample (2 µL) was subjected to UHPLC equipped with a Hypersil GOLD™ VANQUISH™ column (100 × 2.1 mm, particle size: 1.9 µm with a flow rate at 0.4 mL/min). The analysis was started with 5% acetonitrile (ACN): 95% H_2_O for 4 min followed by an increase to 90% ACN: 10% H_2_O for 10 min that was maintained for 4 min. Then, the mobile phase was changed to 5% ACN: 95% H_2_O for 0.5 min, which was maintained for 25 min.

Mass spectral analysis was carried out using Orbitrap in full-scan mode. The mass range was 100–1500 m*/z* with a resolution of 120,000. For dd-MS2 mode, the resolution was 30,000 with collision energies (NCE Stepped) of 10, 30 and 50 for both positive and negative ionization modes.

Data were analyzed using Compound Discoverer 3.1.0.305 software with an untargeted metabolomics workflow. Primary spectra were matched with ChemSpider, Predicted composition, Metabolika Pathway, mzCloud and mzVault databases. Compounds that showed matches with 1–2 databases were tentatively identified. Further confirmation was obtained by fully comparing their secondary mass spectra with the mzCloud, mzVault and an in-house library (mzVault) containing fragmentation patterns of previously analyzed standards. Tentative bioactive compounds were identified by comparing both LC–MS and MS/MS spectra with a minimum of 3 of the aforementioned databases, while also considering literature evidence of their presence specifically in fungi.

### Inhibition of cell-free HMG-CoA reductase by mycelial extract

Inhibition of de novo cholesterol synthesis was determined using an HMG-CoA reductase assay kit (Sigma-Aldrich). Various concentrations of mycelial extract in the range of 0.0125–2.5 mg/mL were mixed with the reaction mixture containing buffer, NADPH, HMG-CoA and HMG-CoA reductase.

Pravastatin provided with the kit was used as a positive control, with DMSO as a negative control (NC). The activity was monitored based on the reduction of NADPH by measuring the absorption at 340 nm at 37 °C every 10 s for 10 min. The activity of every reaction was calculated using the same range of time during the initial velocity of the enzyme shown (activity = ∆A_340_/∆T). Then, inhibition (%) of HMG-CoA reductase was calculated using the following formula:

$$\% \,{\text{Inhibition}}\, = \,\left( {{\text{Activity}}\,{\text{of}}\,{\text{NC}}{-}{\text{Activity}}\,{\text{of}}\,{\text{sample}}} \right)/{\text{Activity}}\,{\text{of}}\,{\text{NC}}\, \times \,{1}00.$$

### Cell culture

The HepG2 cell line was purchased from ATCC and cultured in DMEM supplemented with 10% fetal bovine serum and 1% antibiotic penicillin‒streptomycin. Cells were maintained in a 5% CO_2_ humidified incubator at 37 °C. During subculturing, the cells were detached using trypsinization. The well-grown cells were harvested and seeded into a 96-well plate at a density of 5000 cells per well and incubated for 48 h, and the viability of 2-D HepG2 cells was determined based on the MTT assay. For 3-D spheroid generation, HepG2 cells at 20,000 cells per well were grown on a round-bottomed low-attachment plate. HepG2 spheroids formed and changed in their morphology on Day 5 of culture. After the spheroids had formed, they were treated with lovastatin and the mycelial extract at different concentrations for 48 h. Then, the viability of the spheroids was investigated using the Presto blue assay. The effects of mycelial extract treatment on the biological profile and cholesterol production by the spheroids were investigated after 2 days of incubation.

### Fourier-transform infrared spectroscopy and principal component analysis of HepG2 spheroids

HepG2 spheroids cultured in the control media, 2.5 mg/mL *G. australe* mycelial extracts and 5 µM lovastatin were prepared to perform FTIR analysis. After 48 h of incubation, all samples were washed with cold phosphate buffer saline (PBS), and the supernatant was removed using centrifugation at 230×*g* for 5 min (repeated twice more) followed by DI water for another 3 repetitions. The pellet of spheroids was added to 20 μL DI water and resuspended gently using a pipette tip prior to deposition on a 22 mm diameter × 1.0 mm thickness BaF_2_ window (1–2 μL for each drop) and dried in a desiccator at room temperature until the synchrotron FTIR microspectroscopic experiment commenced at the Synchrotron Light Research Institute in Nakhon Ratchasima, Thailand, using the Infrared Spectroscopy and Imaging BL4.1 unit. The process was carried out using a photon energy range of 0.01–0.5 eV with a 36× Schwarzschild Objective and a Bruker Vertex 70 spectrometer coupled with a Bruker Hyperion 2000 microscope (Bruker Optics) and a 100 µm narrow-band mercury-cadmium-telluride detector cooled using liquid nitrogen. The FTIR spectral data collection was controlled using OPUS 7.8 software (Bruker Optics Ltd.). The samples were analyzed in the infrared spectral range of 4000–800 cm^−1^ in transmission mode with a 10 × 10 µm square aperture. The spectrum of each selected sample area was recorded at 6 cm^−1^ resolution and represented the average of 64 scans. Appropriate FTIR spectra with the amide I band intensity in the range 0.2–1.2 abs were extracted and selected from each measured sample image using the OPUS program. To observe biochemical changes in the three sample groups, Unscrambler X 10.5 software (CAMO Analytics) was utilized for principal component analysis (PCA). Data preprocessing was performed using the Savitzky‒Golay transform derivative (derivative order: 2, polynomial order: 3, smoothing points: 17, left points: 8, right points: 8) and subsequently converted for the spectral regions of 3000–2800 cm^−1^ (lipids) and 1700–1112 cm^−1^ (proteins and nucleic acids) based on extended multiplicative signal correction for further PCA. Triplicates of one-third of the secondary derivatized FTIR spectra from the EMSC data treatment of each sample group were reduced by Unscrambler X prior to integration of the peak area by the OPUS program.

### Cholesterol production by 3-D HepG2 spheroids

The amount of cholesterol was quantitated using a cholesterol assay kit-HDL and LDL/VLDL (Abcam). After 48 h of incubation with lovastatin and the mycelial extract, the spheroids were harvested and washed with cold PBS. Total cholesterol was extracted from the samples by adding 100 µL of cholesterol assay buffer and centrifuged at 4 °C for 10 min at 13,000×*g* using a cold microcentrifuge. The supernatant was collected for total cholesterol measurement. For further high-density lipoprotein (HDL) quantitation, one volume of the supernatant was mixed with 2× precipitation buffer and incubated for 10 min at room temperature. HDL was collected from the supernatant after centrifugation at 2000×*g* for 10 min at room temperature; this step was repeated. Fifty microliters of every sample, including cholesterol standards, was mixed with 50 µL of cholesterol reaction mix containing buffer, cholesterol probes, the relevant enzyme mix and cholesterol esterase. The reaction was carried out at 37 °C for 60 min with protection from light. The tandem absorption levels of cholesterol in the total and HDL fractions were determined at 570 nm. The amount of cholesterol was calculated from the slope of the standard curve, and the fraction of HDL of the total cholesterol from each sample was presented as a percentage. One-way ANOVA was used to analyze the effect of the 2.5 mg/mL extract compared with the control and 5 µM lovastatin.

### Statistical analysis

Three independent experiments from 3 different mycelial cultures were performed for every assay. The results are presented as the mean ± SEM. Statistical analysis was carried out using GraphPad Prism software. Significant differences between samples were tested at *p* < 0.05, as indicated in each result.

## Results

### Identification of *Ganoderma australe*

**Figure 1:**
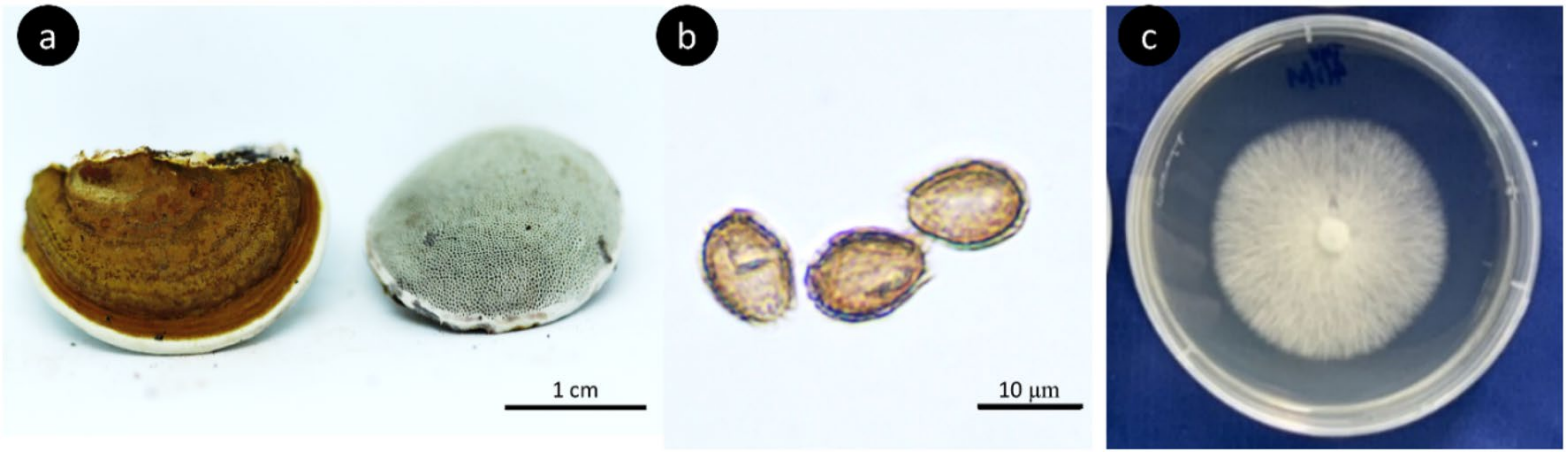
Morphology of *Ganoderma australe* (**a**) Fruiting body. (**b**) Basidiospores. (**c**) Mycelia on PDA plates.

The sample specimen in this study was in the young stage (Fig. 1a) with a pileus 2–2.5 cm long, 1.5–2.0 cm wide and 0.5 cm thick. Pileus flabella formed to subdimidiate. The pileus surface was nonlaccate, slightly soft at the margin and concentrically sulcate at the center toward the margin. The pileus color was brownish-yellow with white at the margin. The context was composed of coarse loose fibrils. The tube was 0.3–0.5 cm long and brown. The stipes were sessile and broadly attached. There were 3–4 pores/mm, subcircular to circular. The pore surface was white. The hyphal system was trimitic; generative hyphae were 1.5–2.0 µm broad, thin-walled, hyaline, tapering at branches with clamp connections; binding hyphae were 1.5–2.5 µm broad, thick-walled and branched; and skeletal hyphae were 3.0–4.0 µm broad, thick-walled and nearly solid. Basidiospores were mostly ellipsoid, 7.3–10 × 4.5–8.7 µm in size, double walled with a brownish-orange inner wall but a dark brown outer wall, with an echinulate truncated end (Fig. 1b).

The mycelia grown on the PDA plates were cottony in a concentric ring (Fig. 1c). The PCR products of 634 bp were subjected to NGS-based barcode taq sequencing. Based on the BLASTN program (NCBI), the sequences of PCR fragments had 99.84% and 99.68% identity with the sequences of *Ganoderma australe* LC084706.1 and KJ654369, respectively. Consistent with the morphology of the fruiting bodies, sequencing data of the mycelia clearly identified the mushroom sample as *G. australe*.

### HPLC analysis of mycelium extracts and lovastatin

**Figure 2:**
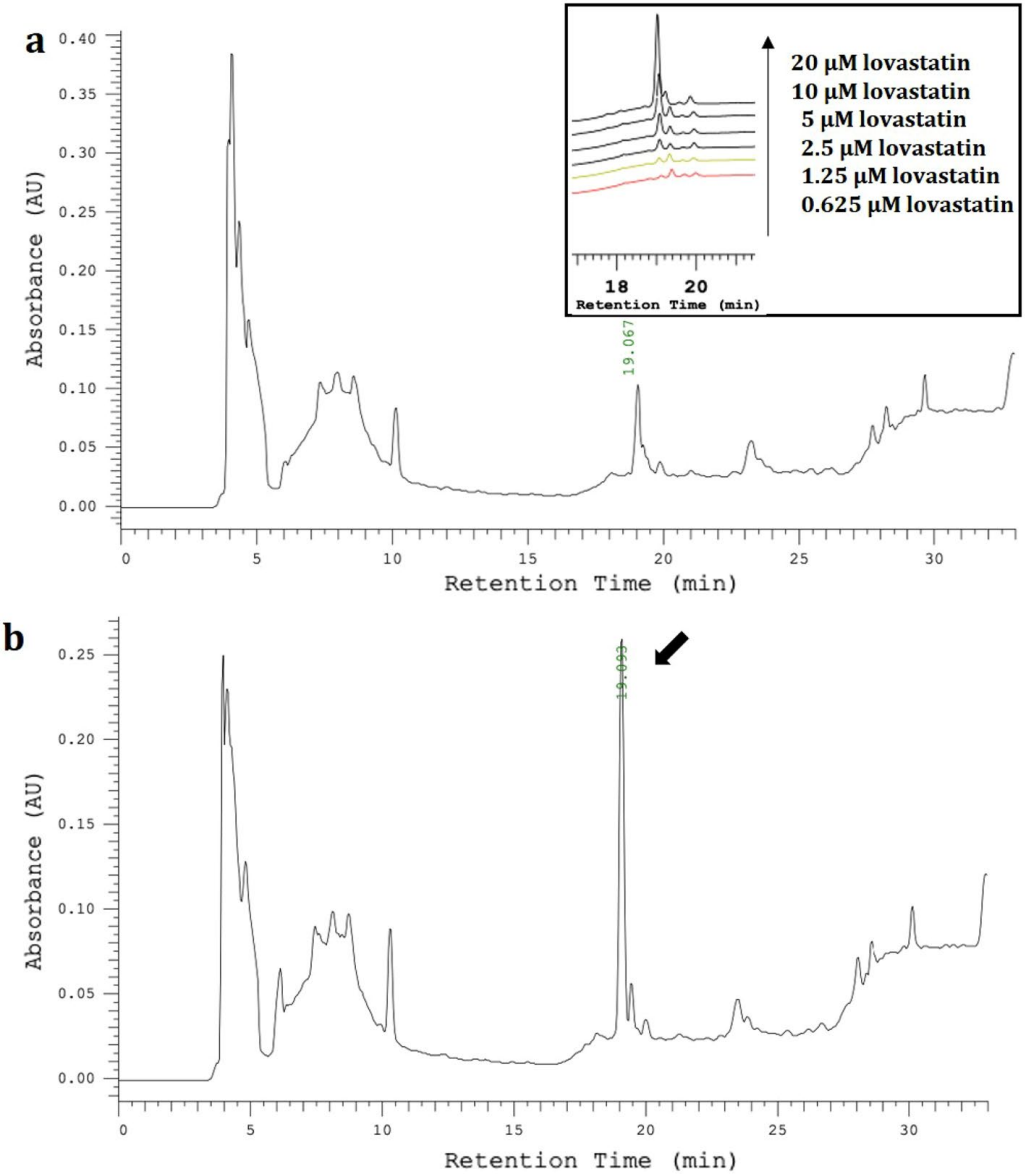
HPLC chromatograms using UV detection at *λ* 240 nm. (**a**) Ten micrograms/mL ethanol extract with inset figure showing varied concentrations of lovastatin standard. (**b**) Spike of 20 µM lovastatin in 8 µg/mL extracts. The black arrow shows persistence in retention time of lovastatin at 19.0 min with increased peak area in spiked samples.

HPLC analysis of the lovastatin standard at different concentrations revealed a retention time of 19.0 min and an LOD of 2.152 × 10^–5^ µM (0.009 ng/mL). With 12.57 ± 2.34% yield of extraction, the three independently cultured mycelial extracts revealed similar chromatographic profiles. Each extraction showed a high peak area with retention times in the range of 4.0–5.5 and 7.0–9.0 min, which appeared to be the major compounds (Fig. 2a), compared to blank control (see Supplementary Fig. S1). The lovastatin standard was spiked into the ethanol extracts to investigate the presence of lovastatin. By comparing the retention times of the extract peaks with the standard, Fig. 2b shows the increased peak height at the exact retention time of lovastatin at 19.0 min (black arrow). Potentially, lovastatin was produced by the mycelia of *G. australe*. Thus, the fraction at a retention time of 19.0 min of the crude extract was collected for further investigation using mass spectrometry techniques.

### Biochemical compositions of *G. australe* mycelial extract

**Figure 3:**
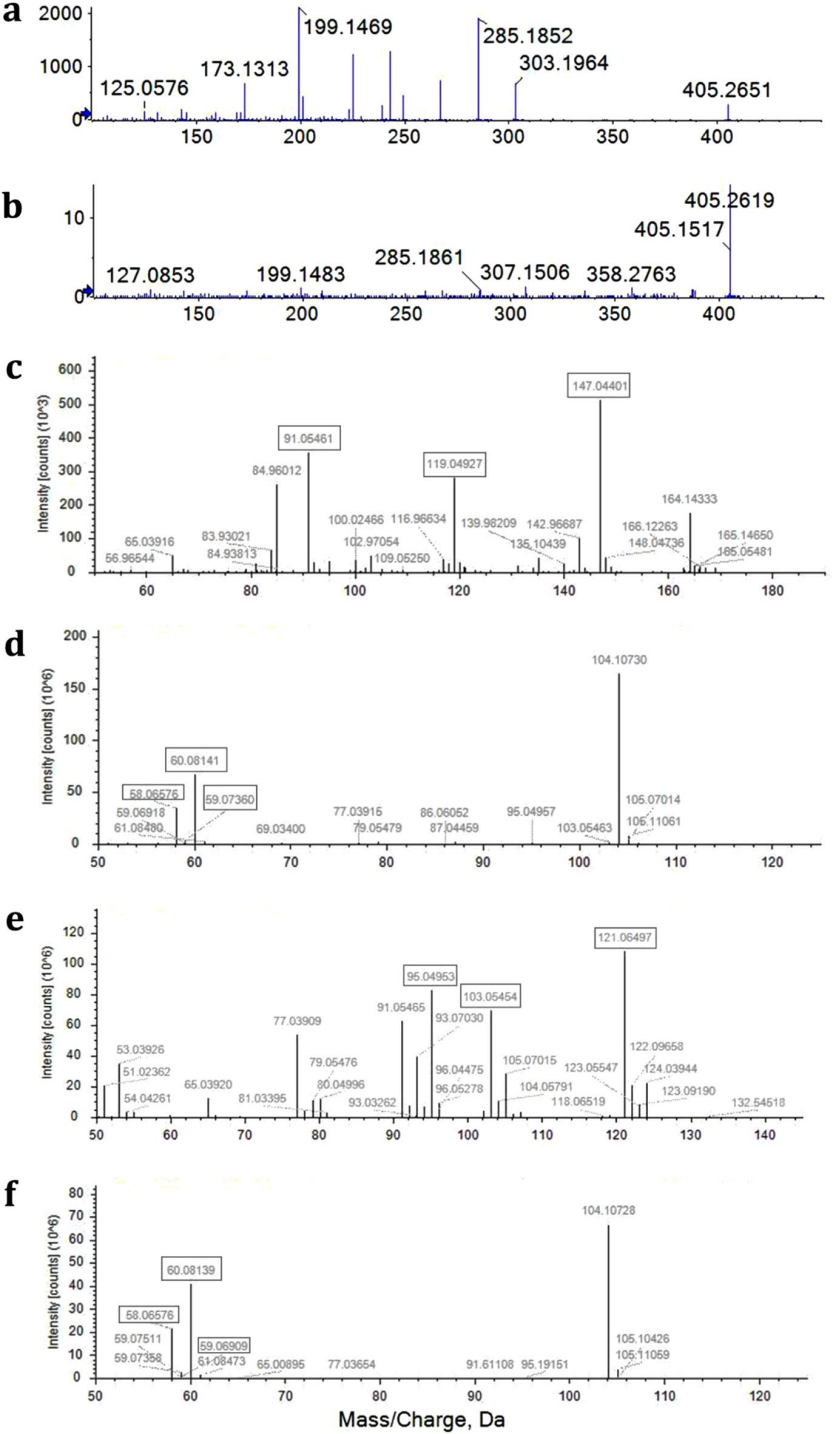
Mass spectral (MS2) patterns. (**a**,**b**) QTOF in MRM mode analysis of (**a**) Lovastatin standard. (**b**) Fractions collected at the same retention time as lovastatin. (**c–f**) Obitrap analysis of crude extract potentially containing (**c**) *p*-coumaric acid. (**d**) Gamma-aminobutyric acid (GABA). (**e**) Nicotinamide. (**f**) Choline. Black frames represent the top three peaks matching fragmentation patterns of tentative compounds in libraries.

ESI–MS in positive mode was used to examine the spectra of the fractions collected at the same retention time as the lovastatin standard. The calculated patterns identified chemical formulas including: (1) C_24_H_36_O_5_ with *m/z* + 1 at 405.2628 and an error of − 0.8 mDa, (2) C_18_H_39_NO_3_ with *m/z* + 1 at 318.2991 and an error of − 1.2 mDa, and (3) C_18_H_30_O_2_ with *m/z* + 1 at 279.2304 and an error of 1.5 mDa. The calculated formulas were potentially those of lovastatin^19^, phytosphingosine^20^ and linolenic acid^21^, respectively, for these components from *Ganoderma* spp. Nevertheless, the intensity of lovastatin was not clear due to noise in the spectral fraction. Thus, the fraction was further examined using HR-MS analysis in MRM mode to acquire the mass spectral pattern that was compared to that of the lovastatin standard (Fig. 3a). The fraction contained 3 prominent peaks at 405.2619, 285.1861 and 199.1483 Da (Fig. 3b) that were matched with lovastatin. Thus, it was concluded that lovastatin was present in the *G. australe* mycelia extracted with ethanol, although in a trace amount of the 3.2 mg of crude extract.

In addition, the ethanol extracts from the mycelia of *G. australe* were investigated for bioactive compounds using LC‒MS/MS methodologies. The results provided by LC/MS Orbitrab revealed the profile of bioactive compounds that have fully or partially, at least the top 3 peaks, matched with fragmentation patterns of tentative compounds in libraries. They have also been reported in other fungi (Table 1), including a phenolic acid, a neurotransmitter, a vitamin, amino acids, saccharides, nucleosides and derivatives. For example, the mass spectral patterns of *p-*coumaric acid, GABA, nicotinamide and choline are shown in Fig. 3c–f, respectively. Moreover, the current investigation identified other derivatives of biomolecules that no other reports of these compounds in fungi were found in the literature (Supplement Data).

**Table 1:** Tentative biocomponents of *Ganoderma australe* extracts identified using LC‒MS/MS analysis.

*m/z* Detected [M + H^+^]	Mass	Formula	Retention time (min)	Tentatively identified biocomponents	Top three fragment mass	MS2-matched library	Classes of compounds
165.0545	164.0472	C_9_H_8_O_3_	8.0310	*p-*coumaric acid	91.05461, 119.04927, 147.04401	In-house library	Phenolic acid
104.0710	103.0638	C_4_H_9_NO_2_	1.590	GABA	58.06577, 59.07360, 60.08139	mzVault	Neuro-transmitter
123.0552	122.0480	C_6_H_6_N_2_O	1.474	Nicotinamide	95.04953, 103.0545, 121.06497	In-house library	Vitamin B3 component
104.1070	103.0998	C_5_H_13_NO	1.583	Choline	58.06576, 59.06909, 60.08139	mzCloud	An essential nutrient
175.1187	174.1114	C_6_H_14_N_4_O_2_	1.546	Arginine	70.06566, 60.05623, 116.0708	mzVault	Amino acids
116.0707	115.0633	C_5_H_9_NO_2_	1.538	Proline	70.0657, 71.06906, 68.05003	mzCloud & mzVault	
132.1018	131.0944	C_6_H_13_NO_2_	1.882	Isoleucine	86.09689, 60.8142, 55.05489	In-house library	
166.0859	165.0786	C_9_H_11_NO_2_	2.016	Phenylalanine	120.0809, 103.0545, 121.0843	In-house library	
132.1016	131.0943	C_6_H_13_NO_2_	1.661	Leucine^a^	88.00475, 115.9644, 99.51267	In-house library	
229.1546	228.1474	C_11_H_20_N_2_O_3_	5.337	Leucylproline	116.0708, 86.09688, 70.0657	mzCloud	
130.0498	129.0424	C_5_H_7_NO_3_	1.593	Pyroglutamic acid	84.04482, 56.05011, 85.02884	mzVault	
118.0863	117.0790	C_5_H_11_NO_2_	1.813	Valine	72.08132, 55.05489, 58.06577	In-house library	
342.1162	341.1089	C_12_H_22_O_11_	1.623	Maltose	203.0527, 185.0422, 185.0422	mzCloud	Mono-, Di-saccharide
182.0784	181.0711	C_6_H_14_O_6_	1.613	l-Iditol	69.03409, 83.04968, 85.02889	mzCloud	
180.0864	179.0792	C_6_H_13_NO_5_	1.564	d-Glucosamine	162.0762, 72.04496, 84.04488	mzCloud	
252.1088	251.1015	C_10_H_13_N_5_O_3_	1.915	2′-Deoxyadenosine	136.0617, 84.04484, 137.0652	mzCloud & mzVault	Nucleosides and derivatives
268.1038	267.0966	C_10_H_13_N_5_O_4_	1.817	Adenosine	136.0618, 137.0652, 250.0922	mzCloud	
113.0346	112.0274	C_4_H_4_N_2_O_2_	1.907	Uracil	70.02932, 96.00842, 95.02443	mzCloud	
269.0879	268.0806	C_10_H_12_N_4_O_5_	1.960	Inosine	137.0458, 110.0716, 136.0618	mzCloud & mzVault	
282.1197	281.1124	C_11_H_15_N_5_O_4_	2.261	2′-O-Methyladenosine	136.0619, 137.0652, 208.8835	mzCloud	
127.0502	126.0429	C_5_H_6_N_2_O_2_	2.112	Thymine	110.0239, 54.03449, 84.04485	mzCloud & mzVault	
137.0457	136.0383	C_5_H_4_N_4_O	1.961	Hypoxanthine	110.0351, 119.0354, 81.07033	mzCloud	
112.0506	111.0434	C_4_H_5_N_3_O	1.634	Cytosine	55.93503, 70.06571, 95.02434	mzCloud & mzVault	
244.0930	243.0854	C_9_H_13_N_3_O_5_	1.884	Cytidine	112.0508, 113.0541, 224.0526	mzCloud & mzVault	
152.0565	151.0492	C_5_H_5_N_5_O	1.937	Guanine	135.0302, 110.0351, 128.0455	mzCloud	
284.0986	283.0914	C_10_ H_13_ N_5_ O_5_	1.937	Guanosine	152.0566, 153.0601, 135.0301	mzVault	
166.0721	165.0649	C_6_H_7_N_5_O	1.775	7-Methylguanine	84.96014, 124.0507, 149.0457	mzCloud	
298.1145	297.1072	C_11_H_15_N_5_O_5_	2.211	7-Methylguanosine	166.0723, 167.0759, 121.0649	mzCloud	
153.0405	152.0332	C_5_H_4_N_4_O_2_	1.939	Xanthine	135.0302, 110.0351, 72.93764	In-house library	

^a^Partial match with at least top three peaks matched with the in-house library.

### Inhibitory effect of mycelial extract on cell-free HMG-CoA reductase

**Figure 4:**
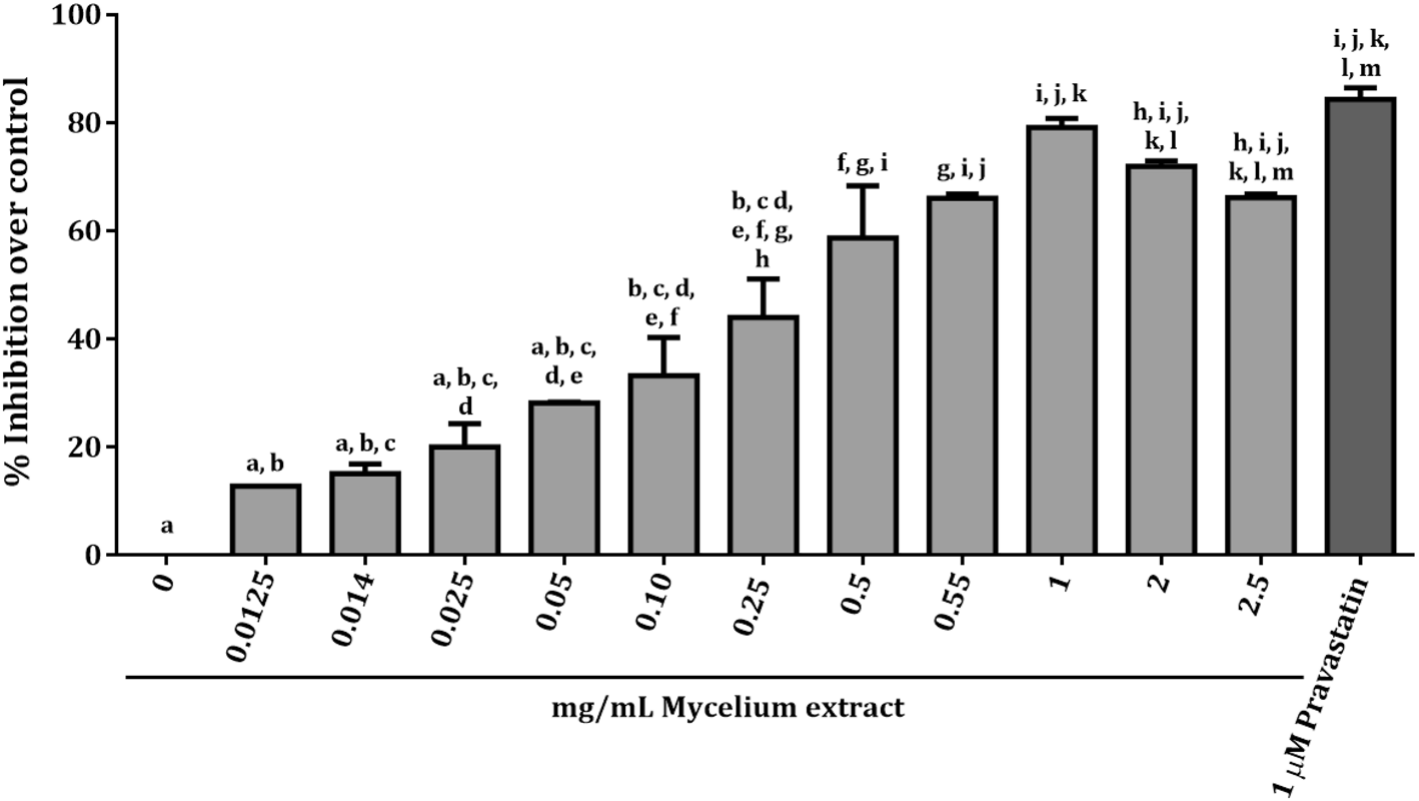
Inhibition of cell-free HMG-CoA reductase activity by *G. australe* mycelial extract. The ethanol extract was dissolved in DMSO in a concentration range of 0.0125–2.5 mg/mL. DMSO was used as a negative control and pravastatin was used as a positive control. The decreased rates of NADPH were calculated from the reduction in spectrophotometric absorbance at 340 nm. The results are presented as the percentage of the negative control in the absence of compounds. Bars indicate the mean ± SEM of triplicate experiments. One-way ANOVA with Tukey's multiple comparisons test to compare with the pravastatin control was performed. Values with letters that do not appear more than once are significantly different from each other (*p* < 0.0001).

Various concentrations of the extract were investigated for their inhibitory effect on the activity of human HMG-CoA reductase (Fig. 4). Enzyme kinetics revealed the highest inhibitory activity of 1 mg/mL extract at 79.08 ± 1.72% over the (negative) control. However, the inhibition was reduced when the concentration of the extract was increased to 2.0 and 2.5 mg/mL, with inhibition percentages of 71.90 ± 1.03% and 66.11 ± 0.72%, respectively. Based on the positive control provided in the kit, 1 µM pravastatin had an inhibition of 84.32 ± 2.10%. Statistical analysis showed that mycelium extracts equal to and higher than 0.50 mg/mL had comparable inhibitory effects to pravastatin. The half-maximal inhibitory concentration (IC_50_) of the mycelial extract was 234.9 ± 1.6 µg/mL. It could be concluded that the mycelial extract had inhibitory activity against cell-free HMG-CoA reductase in a concentration-dependent manner up to 1 mg/mL of the extract. The activity of the extract on cell viability and cholesterol production by liver cells was further examined.

### Effect of *G. australe* mycelial extract on the viability of HepG2 cells

**Figure 5:**
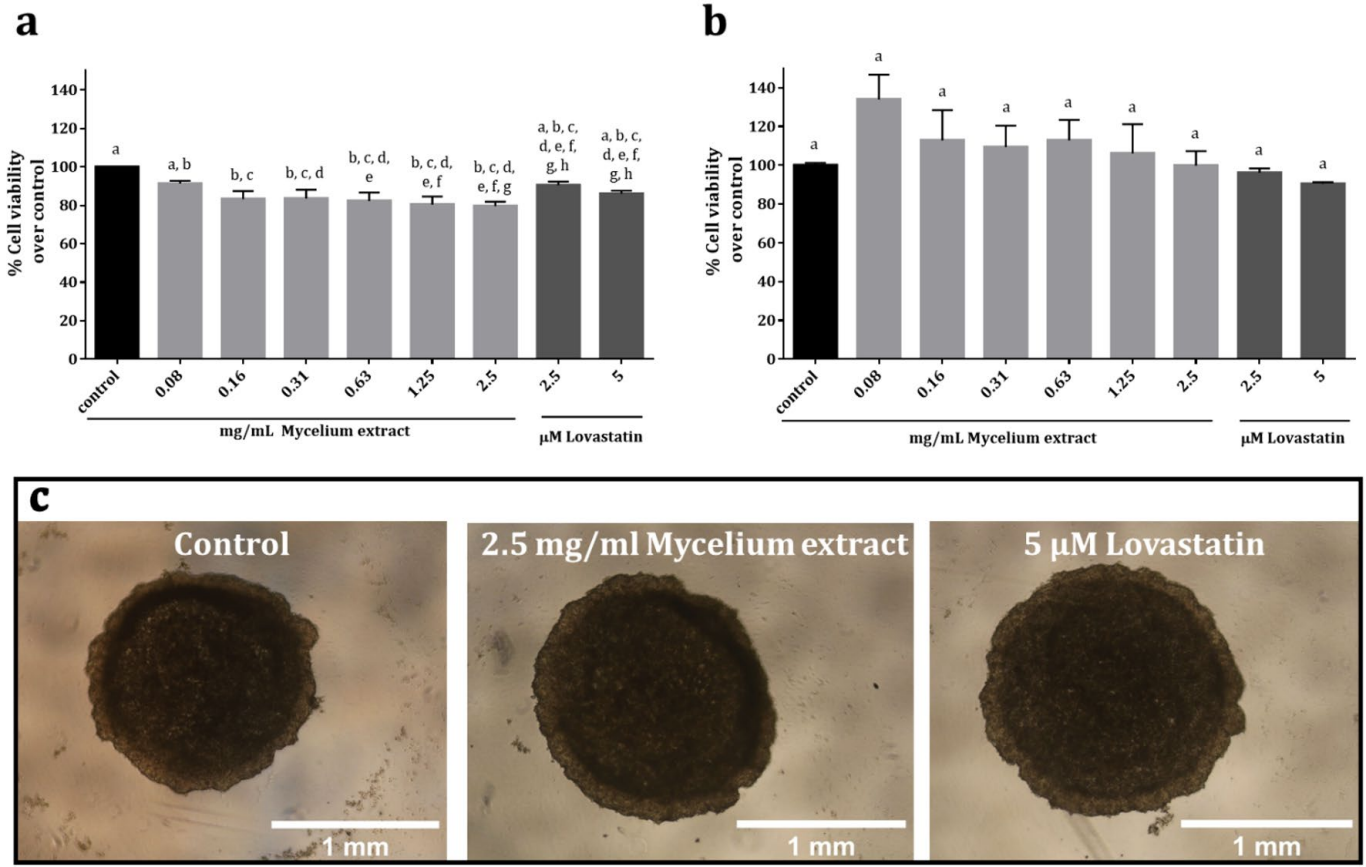
Viability of HepG2 cells and morphology of their spheroids. (**a**) Viability of 2-D HepG2 cells. (**b**) 3-D spheroids presented as percentage of values of cells/spheroids cultured with control media. Cells and spheroids were cultured with various concentrations of the extract and 2.5 and 5 µM lovastatin for 48 h. The results are expressed as means ± SEMs of three individual extractions (*n* = 3), at least in triplicate for each assay. Statistical differences were analyzed using one-way ANOVA with Tukey's multiple comparisons test. Values with letters that do not appear more than once are significantly different from each other (*p* = 0.0004). (**c**) Images of HepG2 spheroids: cultured with media control (left), 2.5 mg/mL mycelial extract (middle) and 5 µM lovastatin (right) for 48 h taken using a light microscope.

HepG2 cells cultured in 2-D and 3-D were used to investigate the effect of mycelial extracts on cell viability. Only concentrations of the extract at 0.08 mg/mL and lovastatin at 2.5 and 5.0 µM did not significantly reduce the viability of 2-D HepG2 cells (Fig. 5a). In contrast, the cell viability of spheroids was not significantly affected by the extract at all concentrations prepared in this experiment or by lovastatin at 2.5 and 5 µM (Fig. 5b). In addition, the shapes of the 3-D HepG2 spheroids were similar and were greater than 1 mm in size for all samples (Fig. 5c). The results showed that the mycelial extract used at 2.5 mg/mL had no spheroid toxicity. Thus, the maximum concentration of the mycelia that could be prepared in this research was used to examine the effect on cholesterol production by spheroids.

### Biochemical profile of HepG2 spheroids analyzed using FTIR

**Figure 6:**
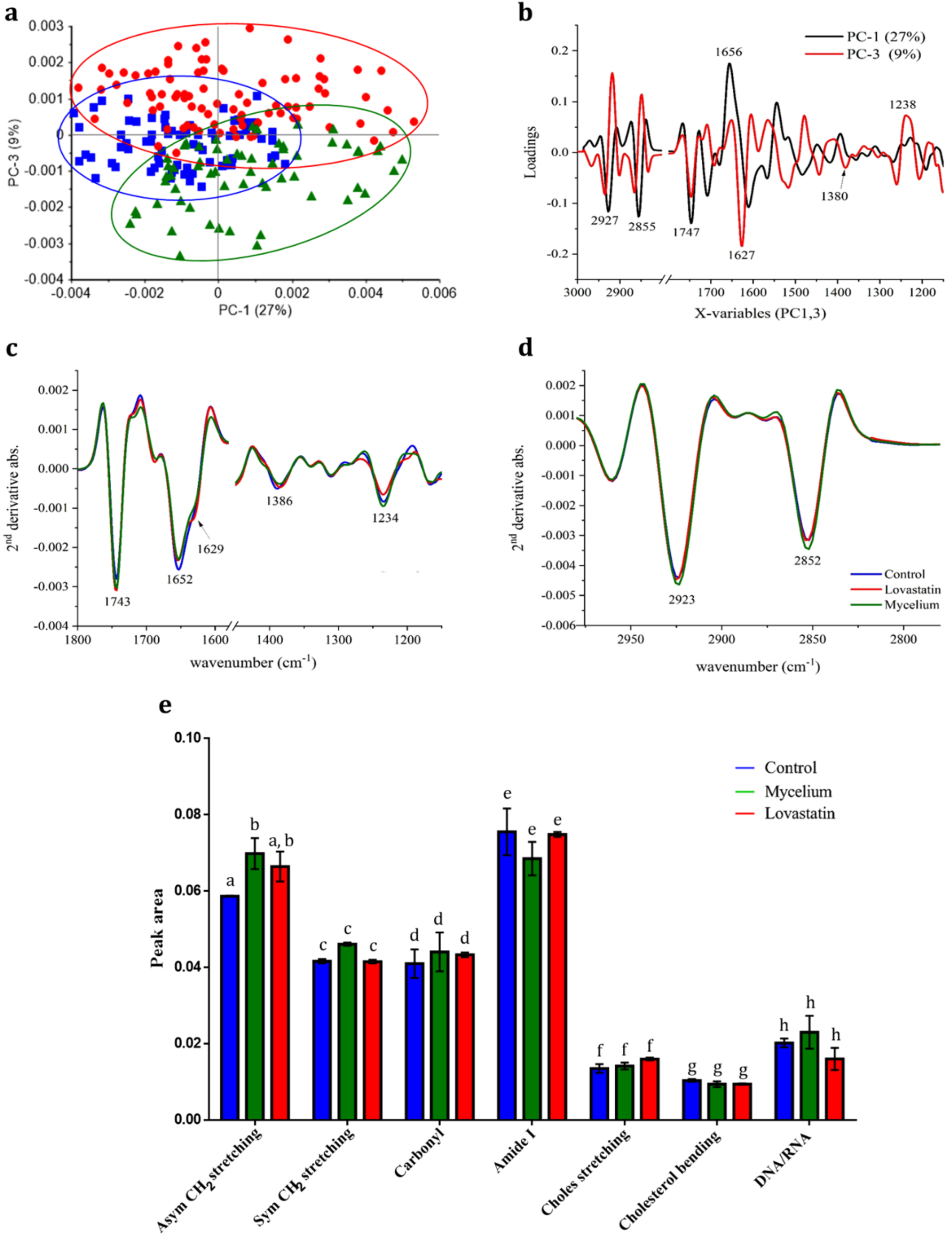
FTIR spectra of HepG2 spheroids. (**a**) Score plot of PC1 and PC3. (**b**) PCA loading plots in the spectral range of 1000–3000 cm^−1^. (**c**) Second derivative spectra of protein and nucleic acid regions in 1000–1700 cm^−1^. (**d**) Lipid regions in 2800–3000 cm^−1^. (**e**) Peak area of secondary spectra. Statistical analysis was performed using two-way ANOVA with Tukey's multiple comparisons test to analyze different functional groups of each sample in triplicate. Values with letters that do not appear more than once are significantly different from each other (*p* < 0.0001). Spheroids treated with control media, 2.5 mg/mL mycelial extract and 5 µM lovastatin for 48 h are presented in blue, green and red, respectively.

The 70, 61 and 83 FTIR spectra of the control, mycelial extract and lovastatin treatment, respectively, were segregated in a 2-D score plot (Fig. 6a). PC-1 (27%) differentiated the control from the mycelial treatment but PC-3 (9%) demonstrated a difference between the lovastatin treatment and the other samples. PCA was used to reveal the variation in the biochemical compounds (Fig. 6b). PC1 and PC3 analysis revealed that the control differentiated from the others by loadings at peaks 2927 and 2855 for lipids and 1656 and 1627 for amide I, while lovastatin was separated from the mycelium by different amounts of cholesterol and nucleic acids at peaks 1380 and 1238 in PC3, respectively. Furthermore, the secondary derivative spectra were analyzed to identify the differentiation of the three major biochemical molecules of proteins and nucleic acids (Fig. 6c) and lipids (Fig. 6d).

The averages of the second derivative spectra in nucleic acid regions (1500–1000 cm^−1^) are shown in Fig. 6c. The three different samples had absorption bands at similar wavenumbers of 1462, 1388, 1235, and 1167 cm^−1^ but with different intensities. The protein region (1700–1500 cm^−1^) presented major absorptions at 1743, 1653, 1633, 1543 and 1516 cm^−1^ (Fig. 6c). Most of these wavenumbers correspond to the vibration of functional groups reported in 2-D HepG2 cells and 3-D human tissue. The wavenumber of 1235 cm^−1^ displayed the asymmetric vibration of phosphodiester bonds of nucleic acids^22,23^. The results showed that the mycelia, control and lovastatin had intensities ranging from high to low. Wavenumber 1743 cm^−1^, which was the stretching of C=O in proteins^24^ and lipids^22,25^, had a similar intensity in the mycelial treatment to the lovastatin treatment but was higher than for the control. Wavenumber 1653 cm^−1^ represented the stretching of the amide I region of the α-helix^22,26^, while 1633 cm^−1^ showed the vibration of the amide I region of the β-sheets in 2-D HepG2^22^ and tissue^27^. The spectral changes from the latter two wavenumbers in the current study indicated that both mycelial- and lovastatin-treated spheroids had reductions in protein in the α-helix structure but conformational change to the β-sheets occurred only in the medicinal-treated samples. All three spheroid samples had approximately the same intensity at 1543 cm^−1^ which was the vibration of amide II^28,29^.

The lipid spectral region at approximately 3000–2800 cm^−1^ demonstrated C–H group vibrations. The spectral average of the three samples displayed high absorbance peaks at 2925 and 2852 cm^−1^ (Fig. 6d). The wavenumber at 2925 cm^−1^ referred to an asymmetric vibration of CH_2_ in lipids^25,30^; 2852 cm^−1^ was the symmetric stretching vibration of CH_2_^25,27^ observed in both 2-D HepG2 and human tissue. The *G. australe* mycelial-treated spheroids had higher intensity peaks at 2852 and 2925 cm^−1^ than the control and lovastatin samples, suggesting a relatively high proportion of lipids.

Figure 6e shows the peak area of the secondary spectra of each sample which had band assignments for asymmetric and symmetric CH_2_ stretching (2936–2912 and 2863–2842, respectively), carbonyl groups (1754–1733), amide I (1670–1619), cholesterol CH_2_ and CH_3_ symmetric stretching (1469–1438) and bending (1404–1368)^31,32^, as well as asymmetric phosphate stretching groups in DNA and RNA (1255–1208). The analysis revealed that only asymmetric CH_2_ stretching of the mycelium extract was significantly higher than that of the control. There was a tendency for symmetric CH_2_ stretching of the mycelium extract and cholesterol stretching of the lovastatin treatment to be higher than those of the other samples. Because cholesterol is a type of lipid and the product of the mevalonate pathway, it and its subtype, HDL, were further quantified.

### Effect of mycelial extract on cholesterol production by HepG2 spheroids

**Figure 7:**
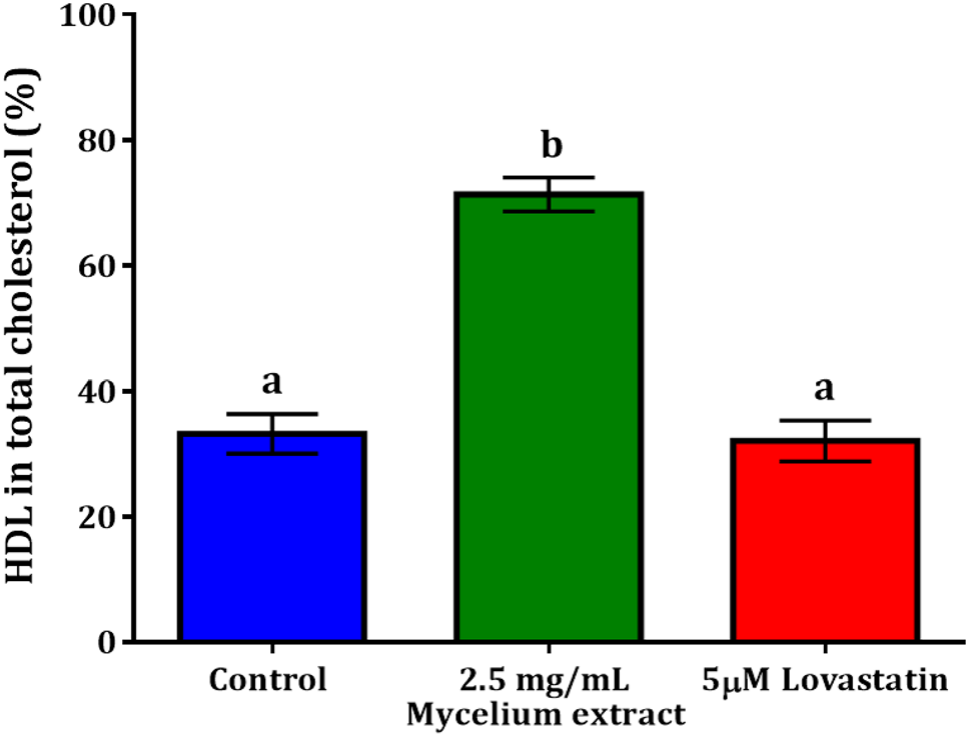
Cholesterol production by HepG2 spheroids. 3-D cells were cultured with control media, 2.5 mg/mL mycelial extract and 5 µM lovastatin for 48 h. HDL content is presented as the percentage of total cholesterol of each individual sample. Bars indicate the mean percentage ± SEM of each sample. Three individual cultured mycelia were investigated in triplicate. Statistical analysis was performed using one-way ANOVA with Tukey's multiple comparisons test. Values with letters that do not appear more than once are significantly different from each other (*p* = 0.0004).

The liver cells were grown in a 3-D spheroidal shape. After 48 h of incubation with 2.5 mg/mL extract and 5 µM lovastatin, the cholesterol produced by the spheroids was de-esterified, and the amount of total cholesterol was measured. Then, HDL was separated from the total cholesterol in all samples, based on its density before it was quantitated. The content of HDL was calculated using the standard curve and presented as a percentage of the total cholesterol for individual samples. The spheroids treated with 2.5 mg/mL mycelial extract for 48 h significantly increased the HDL percentage to 71.35 ± 2.74% compared to the control (33.26 ± 3.15%), as shown in Fig. 7. Surprisingly, not only did 5 µM lovastatin change the HDL percentage of the total cholesterol during incubation reaching an HDL percentage of 32.13 ± 3.24%, but it also increased the amount of total cholesterol to 6.11 ± 0.50 µg. However, the total amount of cholesterol in the mycelial extract and the control was approximately the same (3.32 ± 0.47 µg and 3.25 ± 0.93 µg, respectively). Accordingly, the results indicated the effect of mycelial extraction on increasing HDL production in 3-D liver cell models.

## Discussion

Our morphological identification showed that most characteristics of the specimen were similar to those of *G. australe*, as described by Luangharn et al.^15^, except for the pale green color of the pore surface that was instead concordant with the young fruiting body described by Yamashi and Hirose^33^. The obtained sequence of mycelia covering the ITS1-5.8S-ITS2 sequence region was reported by Bellemain et al.^34^ to potentially be a fungal DNA barcode. Thus, the sequence of this specimen can be clearly used for fungal identification. It showed high similarity to *G. australe* (> 99%) obtained from Southeast Asian countries, including Malaysia (LC084706.1)^33^ and Indonesia (KJ654369). Therefore, the mushroom sample was identified as *G. australe* based on morphological characteristics and molecular identification*.*

*Ganoderma australe* belongs to the genus *Ganoderma*, which has been widely reported for medicinal functions and the production of lovastatin by the mycelia of *G. lucidum*, which was extracted with ethanol^19^, similar to the current work. The identification of lovastatin in the mycelial extract of *G. australe* was initially made by comparing the retention times of sample peaks with the standard compound. The peak area of the crude extract was higher than the LOD of the technique, leading to the spiking method in which the standard solutions of lovastatin were added into the sample as internal standards. The potential presence of lovastatin in the sample showed up as an increase in peak height for the appropriate retention time. Thus, mass analysis was further performed.

The tandem-mass spectral patterns of the fraction originally established the presence of lovastatin in the mycelia of *G. australe* extracted using ethanol, despite containing trace amounts of this compound. Different parts of the mushroom (such as fruiting bodies) and different substrates or fermentation methods could be used to increase the amount of lovastatin produced^35^.

The biocomponent profiles of the *G. australe* mycelia were also scrutinized. Most of the identified compounds have pharmaceutical effects and are found in mushrooms. For example, polysaccharides are present in *Garnoderma* spp., with extensive reporting of their biological activities, such as antioxidant, antitumor and antimicrobial activities^36^. However, only monomers and disaccharides were tentatively found in the extract from the current study, perhaps because less hydrophilic solvent was used in this protocol to extract the bioactive ingredients. Additionally, nucleosides play a role in the maintenance of the immune response and are present in mushrooms^37,38^. The current biocomponent profiles revealed many nucleosides and their derivatives. Palmitic (C16), linoleic (C18) and oleic (C18) acids are the major fatty acids present in the petroleum extraction of the fruiting bodies of *G. australe*^39^. In the current study, only linolenic (C18) acid, an omega-3 lipid, was reported as being potentially present in the mycelia of *G. australe* perhaps because a polar solvent was applied during extraction. The presence of these compounds should be further investigated, compared to their standards. Furthermore, alkaloids^40^ and meroterpenoids^41^ have been recently discovered in the fruiting bodies of *G. australe* extracted using a different organic compound (ethyl acetate). Nicotinamide, GABA and choline are bioessential components in humans and they are potentially found in the mycelia of *G. australe*, as reported in the current work. Furthermore, *p-*coumaric acid is used as a supplement to alleviate hyperlipidemia^42,43^ and was also found in the current work. Thus, it is a potential contributor to the medicinal functions of *G. australe* mycelia.

Triterpenoids are one of the most studied compounds contained in *Garnoderma* spp. Recently, many new lanostane-type triterpenoids have been identified in *G. australe* extracts from mycelia^44^ and fruiting bodies^45–47^ using alcohol and ethyl acetate. Some of these compounds have demonstrated antituberculosis activity, moderate inhibition of nitric oxide production and a significant inhibitory effect on the activity of α-glucosidase. Furthermore, a triterpenoid metabolite, 7-oxo-ganoderic acid Z (C_30_H_46_O_4_) has an inhibitory effect on HMG-CoA reductase^48^, the rate limiting enzyme in de novo cholesterol synthesis. The pure compound extracted from the fruiting bodies of *G. lucidum* with 95% ethanol had an IC_50_ value of 22.3 µM. This leads to the hypothesis that the *G. australe* mycelial extract might have inhibitory activity against HMG-CoA reductase.

Human cholesterol is primarily synthesized on a daily basis by the liver^49^ and controlled by feedback inhibition at the rate limiting step of the pathway that is catalyzed by HGM-CoA reductase. Therefore, any compounds having an inhibitory effect on HMG-CoA reductase activity are of interest for use in hypercholesterolemia treatment. Consequently, in the current study, the effect of the mycelial extract on the inhibition of the human enzyme in vitro, the viability of liver cells and the production of cholesterol by 3-D liver cells was observed. The result from in vitro HGM-CoA reductase assay demonstrated that the *G. australe* mycelial extract at 0.5 mg/mL (50 µg in a reaction) showed 58.69% inhibition, which was higher than the inhibitory effect of 23 medicinal plants at the same amount in the in vitro reaction^50^.

There are bioactive compounds present in *Ganoderma* spp. that have an inhibitory effect on HMG-CoA reductase, such as lovastatin^19^, lanostane triterpenes^51^ and 7-oxo-ganoderic Z^48^. Although lanostanes have been identified in the mycelia^44^ and fruiting bodies^45^ of *G. australe*, none of these works have investigated the inhibitory activity of *G. australe* on the enzyme*.* The current study demonstrated the inhibition of cell-free HMG-CoA reductase in a concentration-dependent manner by *G. australe* mycelial extract. Despite presenting maximum inhibition at 1 mg/mL, the concentration of the crude extract at 2 and 2.5 mg/mL showed slightly decreased inhibition that could have been the result of scattering of the light by particulates and decreasing the absorption value to 340 nm. The presence of lovastatin and tentative *p-*coumaric acid (demonstrated using LC‒MS) suggested such an inhibitory effect on the cholesterol rate-limiting enzyme by the mycelial extract.

The effect of the *G. australe* mycelial extract on the viability of 2-D and 3-D HepG2 was observed prior to the investigations of biochemical profiles and cholesterol production. Although 2.5 mg/mL of the extract reduced the proliferation of monolayer HepG2, this concentration did not affect the proliferation of the cells in 3-D form, perhaps due to the differential absorption of the biocomponents by different cell models; thus, the maximum concentration was used for further analysis. The HepG2 spheroids reflected the three-dimensional form of the liver and physiological functions better than those in monolayer culture. Those functions include detoxification^52^, the production of cholesterol^53^ and the production of apolipoproteins (apoE and apoA-I)^54^. The results suggested the potential use of *G. australe* mycelial extract to examine cholesterol production using a 3-D liver model.

FTIR analysis provided information at the molecular level using vibrational spectra that were specific to the functional groups and bonding types of a molecule. HepG2 spheroids were originally investigated using FTIR in the current work. Most of the obtained spectra were in roughly the same wavenumbers as for 2-D HepG2^22,25,28^ and human tissues representing a 3-D environment similar to this spheroid model^26,27,29,30^. The conformational change in proteins from α-helix to β-sheets that occurred in the lovastatin treatment was also observed in HepG2 cells treated with pravastatin, which is a chemically modified natural statin^22^. In the nucleic acid region of every sample, the similar wavenumbers but different peak intensities indicated the same components despite the different concentrations of nucleic acids. The viability of the treated spheroids suggested that such an effect was unlikely to be from DNA proliferation under laboratory conditions. Thus, altered gene expression potentially occurred.

Overall, FTIR spectroscopy can be used to differentiate the lipid profiles of treated *G. australe* spheroids. It was demonstrated that the mycelial extract increased the amount of lipids produced by HepG2 spheroids, compared to the control and lovastatin. In addition to fatty acids and phospholipids (a constituent of the cell membrane), cholesterol is a type of lipid that is primarily produced by hepatocytes. Thus, vibrations of functional groups of cholesterol were evaluated. Reagent-free LDL and HDL have fingerprint bands at 2852 and 2926 cm^−1^, respectively, for lipid C-H vibration, similar to the current report. Despite being in the protein region, the peaks at 1735 and 1739 cm^−1^ were associated with the vibration of C=O found in reagent-free LDL and HDL, respectively^55^. Those peaks were at 1743 cm^−1^ in the current work but could not be used to differentiate the vibration of the ester of LDL, HDL or protein. The shifted wavenumbers could have come from the different methods of tissue preparation^56^ and the dimension of the samples (for example, 2-D or 3-D of the melanoma cells)^57^.

In the current experiments, the mycelia significantly increased the HDL percentage while maintaining the content of total cholesterol compared to the control. An increased amount of total cholesterol produced by lovastatin incubation was observed in this study, similar to the observed trend of increased peak area around cholesterol stretching in the FTIR result. This could have been due to the effect of homeostatic recovery subsequent to the inhibitory effect of lovastatin, because the activity of HMG-CoA reductase is controlled by feedback inhibition^58^. Comparably, the cholesterol content was increased when HepG2 spheroids were incubated with 1 µM lovastatin for longer than 22 h; however, a reduced amount of cholesterol was observed when the spheroids were treated for a shorter period (4 h) with 10 μM lovastatin^59^.

HDL has become the cholesterol of interest because of its function in reverse cholesterol transportation and its benefit to cardiovascular protection^60^. In such circumstances, the mixture of natural compounds in the *G. australe* mycelial extract increased HDL production by the treated spheroids; notably, this was more effective than pure lovastatin. Further experimentation should be conducted to determine whether this result corresponds with different gene expression and protein production of apoproteins differentially composed of HDL and other cholesterol. For example, apoE and apoA-I (an apoprotein constituent of HDL), which are highly expressed in the spheroid model^54^, should be considered.

Although three-dimensional models have been known to overcome the drawback of monolayer cell culture for mimicking tissue function, further study of the in vivo model should investigate the potential treatment of hypercholesterolemia by functional foods. For example, hydroalcohol mycelial extract^61^ and organic-phase extract^62^ of *G. lucidum* were mixed with food to treat high-fat diet mice at 0.5–1.0% and hamsters at 2.5–5.0%, respectively. The current work used 2.5 mg/mL, comparable to 0.25%, of the mycelium extract to treat HepG2 spheroids, indicating a safe dose for further experiments in animal models. Furthermore, treatment with *p-*coumaric acid, a tentative compound found in the mycelia of *G. australe* in the current work, alleviated the effect of hypercholesterol in vivo^42,43^.

In conclusion, the biocomponents of the *G. australe* mycelial extract were demonstrated for the first time. The extract had an inhibitory effect on the activity of a human cholesterol-rate limiting enzyme, potentially contributed by lovastatin and *p-*coumaric acid in the mycelial extract. The biochemical profiles of the 3-D liver cells changed when treated with lovastatin and the mycelial extract. A distinctive lipid profile was exhibited by the spheroids treated with the mycelium. In addition, the percentage of HDL in the total cholesterol increased compared to the control and pure lovastatin, implying a beneficial effect and suggesting a potential treatment for hypercholesterolemia using natural compound mixtures from *G. australe* mycelia collected in Thailand.

### Supplementary Information

Supplementary Information.

## Data Availability

The authors declare that all data supporting the findings in this research are available within this article.

## References

[1] Nelson RH (2013). Hyperlipidemia as a risk factor for cardiovascular disease. Prim. Care Clin. Off. Pract..

[2] Pirillo A, Casula M, Olmastroni E, Norata GD, Catapano AL (2021). Global epidemiology of dyslipidaemias. Nat. Rev. Cardiol..

[3] Chester A, El Guindy A (2021). From fleming to endo: The discovery of statins. Glob. Cardiol. Sci. Pract..

[4] Ward NC, Watts GF, Eckel RH (2019). Statin toxicity. Circ. Res..

[5] Poli A (2018). Nutraceuticals and functional foods for the control of plasma cholesterol levels. An intersociety position paper. Pharmacol. Res..

[6] Reis FS, Martins A, Vasconcelos MH, Morales P, Ferreira ICFR (2017). Functional foods based on extracts or compounds derived from mushrooms. Trends Food Sci. Technol..

[7] Krittanawong C (2021). Mushroom consumption and cardiovascular health: A systematic review. Am. J. Med..

[8] Wachtel-Galor, S., Yuen, J., Buswell, J. A. & Benzie, I. F. F. *Herbal Medicine: Biomolecular and Clinical Aspects* (eds Benzie, I. F. F., & Wachtel-Galor, S.) (2011). 22593937

[9] Kladar NV, Gavarić NS, Božin BN (2016). Ganoderma insights into anticancer effects. Eur. J. Cancer Prev..

[10] Winska K, Maczka W, Gabryelska K, Grabarczyk M (2019). Mushrooms of the genus ganoderma used to treat diabetes and insulin resistance. Molecules.

[11] Cao Y, Xu X, Liu S, Huang L, Gu J (2018). Ganoderma: A cancer immunotherapy review. Front. Pharmacol..

[12] Chan SW, Tomlinson B, Chan P, Lam CWK (2021). The beneficial effects of Ganoderma lucidum on cardiovascular and metabolic disease risk. Pharm. Biol..

[13] Baby S, Johnson AJ, Govindan B (2015). Secondary metabolites from Ganoderma. Phytochemistry.

[14] Hyde KD (2018). Thailand’s amazing diversity: Up to 96% of fungi in northern Thailand may be novel. Fungal Divers..

[15] Luangharn T (2021). Ganoderma (Ganodermataceae, Basidiomycota) species from the Greater Mekong Subregion. J. Fungi.

[16] Jannual N, Nipitwattanaphon M, Hasin S, Kaewgrajang T (2020). Morphological and molecular characterization of Termitomyces (Lyophyllaceae, Agaricales) in Thailand. Biodivers. J. Biol. Divers..

[17] Altschul SF, Gish W, Miller W, Myers EW, Lipman DJ (1990). Basic local alignment search tool. J. Mol. Biol..

[18] Paemanee A (2022). Mass spectrometry and synchrotron-FTIR microspectroscopy reveal the anti-inflammatory activity of Bua Bok extracts. Phytochem. Anal..

[19] Lo YC (2012). Comparative study of contents of several bioactive components in fruiting bodies and mycelia of culinary-medicinal mushrooms. Int. J. Med. Mushrooms.

[20] Zhang L (2022). Antioxidant, hypoglycemic and protection of acute liver injury activities of Ganoderma lucidum spore water extract. J. Funct. Foods.

[21] Saadoon HF, Khorsheed AC, Abdul-Hadi SY (2021). Analysis of many fatty acid and volatile oils in some *Ganoderma* spp. using gas–liquid chromatography technique. IOP Conf. Ser. Earth Environ. Sci..

[22] Ressaissi A, Pacheco R, Serralheiro MLM (2021). Molecular-level changes induced by hydroxycinnamic acid derivatives in HepG2 cell line: Comparison with pravastatin. Life Sci..

[23] Fukuyama Y, Yoshida S-H, Yanagisawa S, Shimizu M (1999). A study on the differences between oral squamous cell carcinomas and normal oral mucosas measured by Fourier transform infrared spectroscopy. Biospectroscopy.

[24] Ahmed IA, Gai F (2017). Simple method to introduce an ester infrared probe into proteins. Protein Sci..

[25] Khalifa O (2022). Investigation of the effect of exendin-4 on oleic acid-induced steatosis in HepG2 cells using fourier transform infrared spectroscopy. Biomedicines..

[26] Paluszkiewicz C, Kwiatek WM (2001). Analysis of human cancer prostate tissues using FTIR microspectroscopy and SRIXE techniques. J. Mol. Struct..

[27] Fabian H (1995). A comparative infrared spectroscopic study of human breast tumors and breast tumor cell xenografts. Biospectroscopy.

[28] Junhom C, Weerapreeyakul N, Tanthanuch W, Thumanu K (2016). FTIR microspectroscopy defines early drug resistant human hepatocellular carcinoma (HepG2) cells. Exp. Cell Res..

[29] Richter T (2002). Identification of tumor tissue by FTIR spectroscopy in combination with positron emission tomography. Vib. Spectrosc..

[30] Wu JG (2001). Distinguishing malignant from normal oral tissues using FTIR fiber-optic techniques. Biopolymers.

[31] Gupta U, Singh VK, Kumar V, Khajuria Y (2014). Spectroscopic studies of cholesterol: Fourier transform infra-red and vibrational frequency analysis. Mater. Focus.

[32] Vyas PM, Joshi M (2013). Surface micro topographical and dielectric studies of cholesterol crystals. Adv. Mater. Res..

[33] Yamashita S, Hirose D (2016). Phylogenetic analysis of *Ganoderma australe* complex in a Bornean tropical rainforest and implications for mechanism of coexistence of various phylogenetic types. Fungal Ecol..

[34] Bellemain E (2010). ITS as an environmental DNA barcode for fungi: An in silico approach reveals potential PCR biases. BMC Microbiol..

[35] Barrios-Gonzalez J, Perez-Sanchez A, Bibian ME (2020). New knowledge about the biosynthesis of lovastatin and its production by fermentation of *Aspergillus terreus*. Appl. Microbiol. Biotechnol..

[36] Ferreira ICFR (2015). Chemical features of Ganoderma polysaccharides with antioxidant, antitumor and antimicrobial activities. Phytochemistry.

[37] Phan CW (2018). A review on the nucleic acid constituents in mushrooms: Nucleobases, nucleosides and nucleotides. Crit. Rev. Biotechnol..

[38] Yamamoto S, Wang MF, Adjei AA, Ameho CK (1997). Role of nucleosides and nucleotides in the immune system, gut reparation after injury, and brain function. Nutrition.

[39] Martínez AT, Barrasa JM, Prieto A, Blanco MN (1991). Fatty acid composition and taxonomic status of *Ganoderma australe* from southern chile. Mycol. Res..

[40] Zhang JJ, Dong Y, Qin FY, Yan YM, Cheng YX (2021). Meroterpenoids and alkaloids from *Ganoderma australe*. Nat. Prod. Res..

[41] Zhang JJ, Dong Y, Qin FY, Cheng YX (2019). Australeols A-F, neuroprotective meroterpenoids from *Ganoderma australe*. Fitoterapia.

[42] Shen Y (2019). Protective effects of p-coumaric acid against oxidant and hyperlipidemia-an in vitro and in vivo evaluation. Biomed. Pharmacother..

[43] Yoon DS, Cho SY, Yoon HJ, Kim SR, Jung UJ (2021). Protective effects of p-coumaric acid against high-fat diet-induced metabolic dysregulation in mice. Biomed. Pharmacother..

[44] Isaka M (2017). Antitubercular activity of mycelium-associated Ganoderma lanostanoids. J. Nat. Prod..

[45] Guo JC (2021). Triterpenoids and meroterpenoids with alpha-glucosidase inhibitory activities from the fruiting bodies of *Ganoderma australe*. Bioorg. Chem..

[46] Isaka M (2018). Lanostane triterpenoids from cultivated fruiting bodies of the basidiomycete *Ganoderma australe*. Nat. Prod. Res..

[47] Zhou L, Akbar S, Wang MX, Chen HP, Liu JK (2022). Tetra-, penta-, and hexa-nor-lanostane triterpenes from the medicinal fungus *Ganoderma australe*. Nat. Prod. Bioprospect..

[48] Li C, Li Y, Sun HH (2006). New ganoderic acids, bioactive triterpenoid metabolites from the mushroom *Ganoderma lucidum*. Nat. Prod. Res..

[49] Russell DW (1992). Cholesterol biosynthesis and metabolism. Cardiovasc. Drugs Ther..

[50] Baskaran G (2015). HMG-CoA reductase inhibitory activity and phytocomponent investigation of *Basella alba* leaf extract as a treatment for hypercholesterolemia. Drug Des. Dev. Ther..

[51] Wang K (2015). Lanostane triterpenes from the tibetan medicinal mushroom *Ganoderma leucocontextum* and their inhibitory effects on HMG-CoA reductase and alpha-glucosidase. J. Nat. Prod..

[52] Khalil M (2001). Human hepatocyte cell lines proliferating as cohesive spheroid colonies in alginate markedly upregulate both synthetic and detoxificatory liver function. J. Hepatol..

[53] Damelin LH (2004). Altered mitochondrial function and cholesterol synthesis influences protein synthesis in extended HepG2 spheroid cultures. Arch. Biochem. Biophys..

[54] Kurano M (2011). LXR agonist increases apoE secretion from HepG2 spheroid, together with an increased production of VLDL and apoE-rich large HDL. Lipids Health Dis..

[55] Liu KZ, Shaw RA, Man A, Dembinski TC, Mantsch HH (2002). Reagent-free, simultaneous determination of serum cholesterol in HDL and LDL by infrared spectroscopy. Clin. Chem..

[56] Zohdi V (2015). Importance of tissue preparation methods in FTIR micro-spectroscopical analysis of biological tissues: 'Traps for new users'. PLoS ONE.

[57] Srisongkram T, Weerapreeyakul N, Thumanu K (2020). Evaluation of melanoma (SK-MEL-2) cell growth between three-dimensional (3D) and two-dimensional (2D) cell cultures with fourier transform infrared (FTIR) microspectroscopy. Int. J. Mol. Sci..

[58] DeBose-Boyd RA (2008). Feedback regulation of cholesterol synthesis: Sterol-accelerated ubiquitination and degradation of HMG CoA reductase. Cell Res..

[59] Wrzesinski K (2013). HepG2/C3A 3D spheroids exhibit stable physiological functionality for at least 24 days after recovering from trypsinisation. Toxicol. Res..

[60] Ouimet M, Barrett TJ, Fisher EA (2019). HDL and reverse cholesterol transport. Circ. Res..

[61] Meneses ME (2016). Hypocholesterolemic properties and prebiotic effects of Mexican *Ganoderma lucidum* in C57BL/6 mice. PLoS ONE.

[62] Berger A (2004). Cholesterol-lowering properties of *Ganoderma lucidum* in vitro, ex vivo, and in hamsters and minipigs. Lipids Health Dis..

