# Supplementary material for: In vitro cholesterol lowering activity of Ganoderma australe mycelia based on mass spectrometry, synchrotron Fourier-transform infrared analysis and liver-spheroid bioactivity
Source: Sci Rep. 2023 Aug 21;13:13619. doi: 10.1038/s41598-023-40861-8 (PMC10442327; doi:10.1038/s41598-023-40861-8)
Supplement: Supplementary file 1 — Supplementary Information. [file 41598_2023_40861_MOESM1_ESM.docx]

***In vitro* cholesterol lowering activity of *Ganoderma australe* mycelia based on mass spectrometry, synchrotron Fourier**‒**transform infrared analysis and liver**‒**spheroid bioactivity**

Sudthirak Wongkhieo^1^, Wanthongchai Tangmesupphaisan^1^, Jeeraprapa Siriwaseree^1^, Yaovapa Aramsirirujiwet^2^, Prissana Wiriyajitsomboon^2^, Tharnrat Kaewgrajang^3^, Saifa Pumloifa^4^, Atchara Paemanee^4^, Buabarn Kuaprasert^5^, Kiattawee Choowongkomon^1^, Adrian H. Chester^6, 7^, Napachanok M. Swainson^1*^


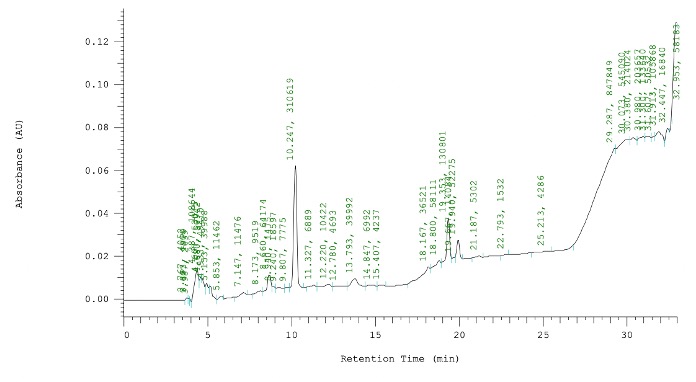
**Supplementary Figure**

**Figure S1.** HPLC chromatograms of blank using UV detection at *λ* 240 nm.

**Supplementary Data**

The current investigation identified modified fatty acid groups, including oleamide (C_18_H_35_NO, mass: 281.2715), palmitoyl ethanolamide (C_18_H_37_NO_2_, mass: 299.2822), (10E, 12Z)-octadecadienoic acid (C_18_H_30_O_3_, mass: 294.2193), and 16-hydroxyhexadecanoic acid (C_16_H_32_O_3_, mass: 271.2277). However, these fatty acid derivatives were not present in the fatty acid profiles of fruiting bodies of *G. australe* that were extracted using petroleum [49]. Derivatives of nitrogenous bases and amino acids were also discovered, including N6-Me-adenosine (C_11_H_15_N_5_O_4_, mass: 190.0708), 8-hydroxy-deoxyguanosine (C_10_H_13_N_5_O_5_, mass: 284.0986), 2'-O-methylguanosine (C_11_H_15_N_5_O_5_, mass: 297.1072), 2'-O-methyladenosine (C_11_H_15_N_5_O_4_, mass: 281.1124), N-acetyl-L-glutamic acid (C_7_H_11_NO_5_, mass: 190.0708), L-saccharopine (C_11_H_20_N_2_O_6_, mass: 277.1392), N-acetyl-L-leucine (C_8_H_15_NO_3_, mass: 173.1051), glycylproline (C_7_H_12_N_2_O_3_, mass: 172.0846) and DL-homoserine (C_4_H_9_NO_3_, mass: 119.0582). A molecule with the formula C_30_H_46_O_4_ and mass 470.3394 Da (m/z 471.3467) was also matched in our library with a unique name suggesting a triterpenoid structure. Its matched name is (2S, 4aS, 6aS, 6bR, 10S, 12aS, 14bS)-10-hydroxy-2,4a,6a,6b,9,9, 12a-heptamethyl-13-oxo-1, 2, 3, 4, 4a, 5, 6, 6a, 6b, 7, 8, 8a, 9, 10, 11, 12, 12a, 12b, 13, 14b-icosahydropicene-2-carboxylic acid, based on our library. However, no other reports of these compounds in fungi were found in the literature.

**Reference**

49 Martínez, A. T., Barrasa, J. M., Prieto, A. & Blanco, M. N. Fatty acid composition and taxonomic status of Ganoderma australe from southern chile. *Mycological Research* **95**, 782-784, doi:<https://doi.org/10.1016/S0953-7562(09)80038-5> (1991).
